# Pharmacokinetics of Nivolumab and Erythropoietin in a Rat Model of Diet-Induced Obesity

**DOI:** 10.1007/s11095-025-03819-1

**Published:** 2025-01-23

**Authors:** Yi-Hua Sheng, Celine Park, Yae Eun Chong, Christine Yohn, Anna Siemiątkowska, Katarzyna Kosicka-Noworzyń, Amrit Kaur, Karan Sapra, Luigi Brunetti, Leonid Kagan

**Affiliations:** 1 Department of Pharmaceutics, Ernest Mario School of Pharmacy, Rutgers, The State University of New Jersey, 160 Frelinghuysen Road, Piscataway, NJ 08854 USA; 2 Department of Pharmacy Practice and Administration, Ernest Mario School of Pharmacy, Rutgers, The State University of New Jersey, 160 Frelinghuysen Road, Piscataway, NJ 08854 USA; 3 Center of Excellence for Pharmaceutical Translational Research and Education, Ernest Mario School of Pharmacy, Rutgers, The State University of New Jersey, 160 Frelinghuysen Road, Piscataway, NJ 08854 USA; 4 Department of Physical Pharmacy and Pharmacokinetics, Poznan University of Medical Sciences, Rokietnicka 3, 60-806 Poznań, Poland

**Keywords:** biologics, body composition, monoclonal antibody, protein therapeutics, subcutaneous absorption

## Abstract

**Purpose:**

To investigate how obesity affects the pharmacokinetics of biologics in a rat model.

**Method:**

Male Long-Evans rats were fed a high-fat diet from the age of 3 weeks and development of obesity was monitored by measuring body size and composition (fat and lean mass). The animals received nivolumab (1 and 8 mg/kg) or recombinant human erythropoietin (rHuEPO, 1000 IU/kg) by intravenous or subcutaneous injection. Serum samples were collected and analyzed using an enzyme-linked immunosorbent assay (ELISA). Endogenous rat IgG was also measured in the nivolumab study. A standard noncompartmental analysis was performed to calculate pharmacokinetic parameters.

**Results:**

When dosed at mg/kg of total body weight approach, no significant differences in pharmacokinetics of nivolumab and rHuEPO between lean and obese cohorts were observed despite significant differences in the body composition. Subcutaneous bioavailability of nivolumab was inversely dependent on the dose level.

**Conclusions:**

Pharmacokinetic parameters of two biologics tested in this work were not affected by obesity, and mg/kg dosing approach was necessary to achieve equivalent exposure in serum. The results were different from our previous findings of significant effect of obesity on pharmacokinetics of human IgG in rats. Additional studies with other biologics are urgently needed in preclinical and clinical settings.

## Introduction

According to National Health Statistics Reports published in 2021, the prevalence of obesity (body mass index ≥ 30 kg/m^2^) in adults in the United States was 41.7% [1]. Obesity is a chronic multisystem disease associated with multiple comorbidities including cardiovascular and metabolic diseases and various cancers [2]. Managing these comorbidities often requires multiple medications. However, our understanding of how obesity affects pharmacokinetics is limited, and appropriate dosing strategies for treating patients with obesity are often lacking (especially for biologics).

Biologics are the largest class of drugs and are used in a variety of conditions, including many forms of cancer. Many biologics are dosed according to body size measurements such as total body weight, adjusted body weight, and body surface area [3, 4]. Such an approach was shown to reduced interindividual variability [5]. However, current trends increasingly favor flat dosing to reduce healthcare costs without compromising efficacy [6, 7]. For example, pembrolizumab and nivolumab were initially approved for weight-based dosing but have now received approval for fixed dosing [8]. Simulation studies also showed that a fixed dosing approach achieves pharmacokinetic profiles comparable to a body-size-dependent dosing approach for some biologics [5, 9]. In our previous preclinical study, the area under the serum concentration-time curve for human IgG was lower and the clearance was higher in obese rats compared to control animals after receiving intravenous or subcutaneous administration of human IgG at a dose level of 1 g/kg of total body weight [10]. Furthermore, in a pilot human study, the half-life of IgG negatively correlated with subjects’ body mass index and fat mass [11]. These studies suggest that further research is needed to identify optimal dosing strategies for IgG-based therapies in obesity.

Two model drugs were selected for this study: an IgG4-based monoclonal antibody (nivolumab) and recombinant human erythropoietin (rHuEPO). These biologics were selected as representatives of two distinct groups of protein drugs: a large molecular weight monoclonal antibody that has a long circulation half-life due to FcRn-mediated recycling and a hormone with a smaller molecular weight and much shorter circulating half-life.

Nivolumab, a programmed death-1 immune checkpoint inhibitor, was first approved by the FDA in 2014 and has been used to treat various cancers including non-small cell lung cancer, melanoma, advanced renal cell carcinoma, esophageal or gastroesophageal junction cancer [12, 13]. Nivolumab is administered intravenously as a monotherapy with a fixed dose of 240 mg every two weeks or 480 mg every four weeks. However, a body weight-adjusted dose is adopted when combined with ipilimumab. According to the label for OPDIVO® (nivolumab), there is currently no recommended dose adjustment for obese population [14]. In some studies, BMI and body composition metrics including skeletal muscle index and subcutaneous adipose tissue index were associated with clinical outcomes or toxicity [15–17]. However, studies directly comparing nivolumab pharmacokinetics between patients with normal weight and patients with obesity have not been published.

Recombinant human erythropoietin (rHuEPO), a 30-kDa protein therapeutic drug, was approved by the FDA in 1989, and it is being used to treat anemia due to chronic kidney disease or chemotherapy [18]. According to the label of PROCRIT® (epoetin alfa), the recommended starting dose for adult patients with chronic kidney disease is 50–100 units/kg 3 times weekly by intravenous or subcutaneous administration; the recommended starting dose for adult patients with chemotherapy-induced anemia is 150 units/kg 3 times weekly subcutaneously or 40,000 units weekly subcutaneously. No specific instruction for dosage adjustment in obesity are provided on the label [19].

The aim of the study was to compare the pharmacokinetics of nivolumab and rHuEPO after intravenous and subcutaneous administration between rats with diet-induced obesity and lean controls.

## Materials and Methods

### Materials

Nivolumab (OPDIVO®; 10 mg/mL) was obtained from Rutgers Cancer Institute of New Jersey (New Brunswick, NJ). Recombinant human erythropoietin (PROCRIT®; 10,000 units/mL) and Isoflurane inhalation solution 99.9% was purchased from Henry Schein (Melville, NY). Blank rat serum was from BioIVT (Hicksville, NY). Goat anti-human IgG antibody (SAB3701327), goat anti-human IgG (Fc specific) − peroxidase antibody (A0170), bovine serum albumin (BSA), and o-phenylenediamine (OPD) tablets were purchased from MilliporeSigma (Burlington, MA). Carbonate-bicarbonate buffer packs, polysorbate 20 (Tween-20), clear flat-bottom 96-well plates, phosphate buffered saline (10X PBS), and Invitrogen human erythropoietin sandwich ELISA kit (BMS2035-2) were ordered from Thermo Fisher Scientific (Waltham, MA). Rat IgG SimpleStep® ELISA kit (ab189578) was purchased from Abcam (Cambridge, MA). Sulfuric acid (2 N) was from Ricca Chemical Company (Arlington, TX).

### Animals

The animal study was conducted according to an approved protocol from the Institutional Animal Care and Use Committee (IACUC) of Rutgers, the State University of New Jersey. Male Long-Evans rats were purchased from Envigo (Indianapolis, IN). Initially, rats were housed at an Envigo animal facility, where they had free access to water and standard diets for the first three weeks after birth. At the age of 3 weeks, the rats were randomized into two groups and fed either the Teklad high-fat diet (HFD; Custom Diet TD.06414) or the Teklad regular diet (2014S). At the age of 11–12 weeks, the rats were transferred to the Rutgers animal facility, where they were housed in pairs, maintained on a 12/12 h dark/light cycle, and continued on either high-fat or regular diets until the end of the study. Animals were under light isoflurane anesthesia during drug administration and sample collection.

### Measurement of Body Metrics and Composition

The body size measurements were performed, including total body weight (TBW), abdominal circumference (AC), body length, and body compositions. For the nivolumab study, TBW was measured at the age of 3 and 7 weeks; TBW and body compositions were measured at the age of 12 weeks to ensure sufficient separation between the lean and obese groups before the dosing day. All the body measurements were taken weekly from the dosing day until the end of the study. In the rHuEPO study, body measurements were taken at 18 weeks of age to ensure sufficient separation between the lean and obese cohorts, with an additional measurement taken just before dosing.

Body composition was measured using EchoMRI™ analyzer (EchoMRI, Houston, TX). The body composition analyzer was calibrated first using a clear cylinder tube filled with canola oil (according to the manufacturer’s instructions). The body composition of rats was measured without anesthesia; each animal was kept in a cylinder holder for approximately 2 min for a scan that provided measurement of fat and lean mass. Fat mass is defined as a collective mass of all adipose tissues, and lean mass is the mass of all water-containing tissues except for bone, fat, and tissues that do not generate signals in nuclear magnetic resonance imaging, such as hair and claws (http://www.echomri.com).

### Study Design

A diet-induced obesity rat model was established by feeding animals with a high-fat diet. Dosing was initiated once a sufficient separation of body metrics (double amount of fat mass in obese *vs*. lean cohort) was achieved from the control lean animals (fed with a standard diet).

In the nivolumab study, the animals were 16 weeks old when dosing. Each cohort (lean or obese) was divided into 4 subgroups (n = 5–6 each) according to the route of administration and the dose level. The animals were given nivolumab at 1 or 8 mg/kg of total body weight by intravenous (IV) or subcutaneous (SC) injection. For IV injection, nivolumab was administered via the tail vein and followed with 200 μL of normal saline to guarantee full delivery of IV dose. For SC administration, nivolumab was injected in the middle of the abdomen. Serial blood samples (100 μL) were collected from the saphenous vein at 20 min, 1, 3, 5, 8 h and 1, 2, 4, 7, 9, 14, 21, 35 days after IV dosing; and at 1, 3, 5, 8, 10 h and 1, 2, 3, 4, 7, 9, 14, 21, 35 days after SC dosing.

In the rHuEPO study, the animals were 19–21 weeks old at the time of dosing. Each cohort (lean or obese) was divided into 2 groups (*n* = 8 each) according to the route of administration. Animals received rHuEPO at 1000 IU/kg of total body weight IV (via the tail vein) or SC (in the middle of the abdomen). Due to the relatively large blood volume needed for the bioanalytical assay (200 µL), each group was subdivided into two subgroups. For the IV administration, in one subgroup (*n* = 4) blood samples were collected from the saphenous vein at 10 min, and then at 1, 4, 12, and 32 h after dosing, and in the second subgroup (*n* = 4) samples were collected at 30 min, and then at 2, 8, 24, and 48 h after dosing. For the SC administration, in one subgroup (*n* = 4) blood was collected at 1, 4, 12, 32 and 72 h after dosing, and in the second subgroup (*n* = 4) samples were collected at 2, 8, 24 and 48 h post-dosing after dosing.

Blood samples were allowed to clot at room temperature for 30–60 min and then centrifugated at 4000 rpm for 5 min to separate serum. Serum samples were aliquoted and stored at − 80°C for further bioanalysis.

### Bioanalytical Assay

A sandwich enzyme-linked immunosorbent assay (ELISA) was developed to determine nivolumab concentrations in serum samples and provided sufficient accuracy and precision. Briefly, a clear flat-bottom 96-well plate was coated with 100 μL of goat anti-human IgG antibody solution (1 μg/mL in 0.2 M carbonate-bicarbonate buffer, pH 9.4) overnight at 4°C. On the next day, the plate was washed 3 times using 250 μL of a washing buffer (0.05% Tween-20 in 1X PBS) and incubated for 1 h at room temperature with 150 μL of a blocking solution (1% BSA in 1X PBS) to prevent non-specific binding. The same washing method was applied between every step afterwards and all the incubations were performed at room temperature except for the coating step. After the blocking step, 100 μL of nivolumab standards in triplicate or serum samples in duplicate were then added to the well and incubated for 1 h. For detection, goat anti-human IgG (Fc specific) − peroxidase antibody (1:50,000 dilution in PBS, 100 μL) was added for 1 h; and plates were developed using 100 μL of freshly prepared OPD for 33 min in the dark. The reaction was stopped using 50 μL 1 M H_2_SO_4_ and the optical density at 492 nm was measured within 10 min. The working range of ELISA was 0.4–500 ng/mL. Different dilution factors were needed for serum samples from different dosing groups: 1:10,000 for IV 10 mg/kg, 1:1,000 for IV 1 mg/kg and SC 8 mg/kg, and 1:100 for SC 1 mg/kg. The samples were diluted with a solution of 1% BSA in 1X PBS.

Endogenous rat IgG was measured using an Abcam rat IgG ELISA kit following the manufacturer’s instructions. The calibration curve had a working range of 0.3125—20 ng/mL, and serum samples were diluted 1:1,000,000 in accordance with the protocol’s suggestions for serum sample dilution.

Quantitation of rHuEPO in rat serum samples was performed using Invitrogen human erythropoietin sandwich ELISA kit according to manufacturer’s instructions. The working range of the assay was 1.6—100 mIU/mL. The serum samples from the IV groups were diluted 100 times while the serum samples from the SC groups were diluted 40 times using blank rat serum.

### Data Analysis

Measurement of body, size, body composition, and serum concentration data for each group were summarized as mean ± standard deviation (SD). Noncompartmental analysis of pharmacokinetic data was conducted using Phoenix WinNonlin 8.3 (Certara, Princeton, NJ) for each individual rat serum concentration-time profile. The maximum serum concentration (C_max_) and the time to reach C_max_ (T_max_) were directly observed from the experimental concentration-time profiles for groups that received the drugs by SC injection. The area under the serum concentration-time curve from time zero to infinity (AUC_inf_) and terminal half-life (T_1/2_) were calculated using the linear up/log down method). Systemic clearance (CL) and volume of distribution (V_ss_) for IV groups or clearance and volume normalized by the bioavailability (CL/F and V_ss_ /F) for SC groups were calculated accordingly. The bioavailability for SC groups was calculated by dividing individual AUC_inf_ values by the mean AUC_inf_ from IV groups of the same dose level.

To compare the data between lean and obese groups of the same route of administration and dose level, two-tailed t-test was performed. A *p* value of less than 0.05 was considered statistically significant. In addition, for nivolumab study, one-way ANOVA was conducted to compare pharmacokinetic parameters across all IV groups and across all SC groups. For ANOVA, AUC_inf_ and C_max_ were dose-normalized. A post-hoc analysis using Tukey's multiple comparisons test was performed after the ANOVA to identify the specific groups that differed from one another.

## Results

**Fig. 1:**
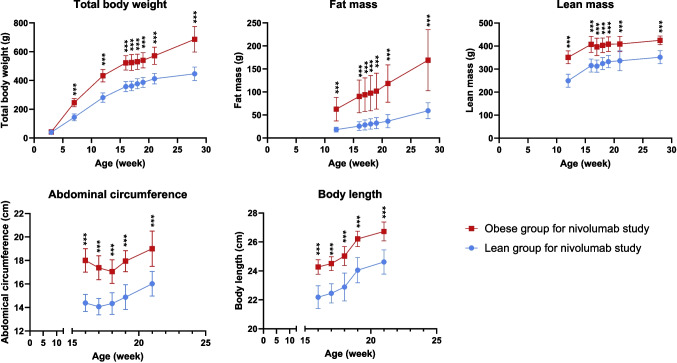
Body size and composition measurements of lean and obese rats in nivolumab study. Data are shown as mean ± SD (*n* = 22 each group). The lean and obese rats were randomly assigned to IV and SC dose groups for nivolumab administration. ***—significant difference (t-test, *p* < 0.001).

**Fig. 2:**
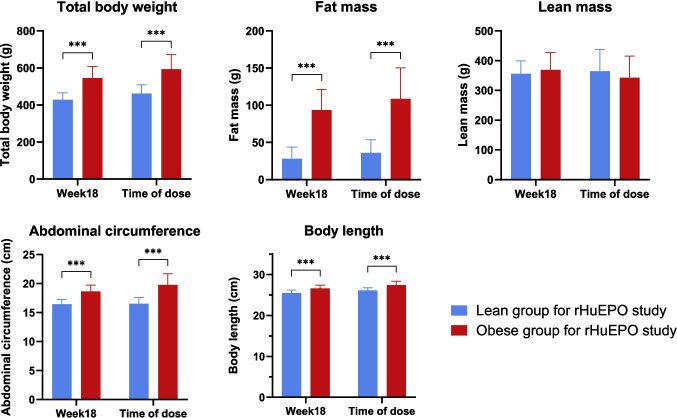
Body size and composition measurements of lean and obese rats in rHuEPO study. Data are shown as mean ± SD (*n* = 16 per group for week 18; *n* = 4 per group for time of dose). The lean and obese rats were randomly assigned to IV and SC dose groups for rHuEPO administration. ***—significant difference (t-test, *p* < 0.001).

Obesity was developed successfully using the high-fat diet as shown in Figs. 1 and 2. In the nivolumab study (Fig. 1), total body weight (TBW) showed significant differences between lean and obese groups at the age of 7 weeks and through the end of the study. At the time of dosing TBW was 522 ± 50 g and fat mass was 90 ± 36 g in the obese group; TBW was 358 ± 35 g and fat mass was 26 ± 10 g in the lean group. Fat and lean masses showed significant differences at the age of 12 weeks, which was maintained until the end of the study. Abdominal circumference (AC) and body length were measured at the beginning of the pharmacokinetics study (at the age of 15 weeks) and significant differences were observed between lean and obese groups throughout the whole study.

In the rHuEPO study as shown in Fig. 2, body measurements were taken when the rats were 18 weeks old to ensure sufficient separation between lean and obese animals. An additional measurement was conducted on the dosing day, when the rats were between 19 and 21 weeks old. At the time of dosing, TBW was 593 ± 80 g and fat mass was109 ± 42 g for the obese group, while the lean group had a TBW of 462 ± 48 g and fat mass of 36 ± 18 g. Statistically significant differences in TBW, fat mass, abdominal circumference, and body length were observed between obese and lean groups at 18 weeks and on the dosing day.

**Fig. 3:**
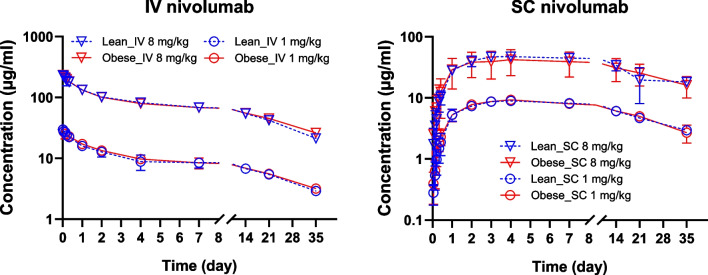
Observed serum pharmacokinetic profiles of nivolumab in obese and lean rats following IV or SC administration of 1 mg/kg or 8 mg/kg. Data are shown as mean ± SD (*n* = 5–6 per group).

Observed mean (± SD) pharmacokinetic profiles of nivolumab in obese and lean rats following IV or SC administration of 1 mg/kg or 8 mg/kg dose levels are shown in Fig. 3. The pharmacokinetic profiles were superimposable between lean and obese groups for two routes of administration and the corresponding dose levels. Table I summarizes pharmacokinetic parameters calculated using a noncompartmental approach. No statistical difference between lean and obese groups at corresponding dose levels and routes of administration was observed based on t-tests. One-way ANOVA was used to compare the parameters within the IV and SC groups separately. For ANOVA, AUC_inf_ and C_max_ were normalized by the dose. The ANOVA and post-hoc results indicated differences of T_1/2_ between IV 1 mg/kg in the obese group and IV 8 mg/kg in the lean group. Furthermore, significant difference was found for SC groups between two dose levels, including dose-normalized C_max_ and AUC_inf_, and bioavailability, which indicated dose-dependent SC absorption of nivolumab.

**Table I:** Pharmacokinetic Parameters of Nivolumab Following Intravenous or Subcutaneous Administration to Lean and Obese Rats Calculated by Noncompartmental Analysis

		IV 1 mg/kg		IV 8 mg/kg		SC 1 mg/kg		SC 8 mg/kg	
Parameter	Units	Lean	Obese	Lean	Obese	Lean	Obese	Lean	Obese
C_max_	μg/mL	-	-	-	-	9.2 ± 0.9^b,c^	8.8 ± 0.7^d,e^	49.0 ± 6.2^b,d^	42.7 ± 19.1^c,e^
T_max_	day	-	-	-	-	3.6 ± 0.5	4.0 ± 0.0	4.8 ± 2.4	3.5 ± 0.6
AUC_inf_	day∙μg/mL	308 ± 37	323 ± 45	2372 ± 207	2637 ± 320	275 ± 19^f^	256 ± 50	1266 ± 541^f^	1493 ± 568
CL or CL/F	mL/day/kg	3.3 ± 0.4	3.1 ± 0.4	3.4 ± 0.3	3.1 ± 0.4	3.7 ± 0.3	4.0 ± 0.7	7.8 ± 4.5	6.2 ± 3.1
V_ss_ or V_ss_/F	mL/kg	80.0 ± 12.0	86.2 ± 16.8	76.6 ± 12.9	81.5 ± 11.2	103.8 ± 16.2	93.8 ± 17.2	112.3 ± 34.9	208.6 ± 125.3
T_1/2_	day	16.9 ± 1.6	18.9 ± 1.6^a^	15.6 ± 1.6^a^	18.4 ± 2.3	19.6 ± 1.8	16.8 ± 5.3	13.6 ± 8.4	22.5 ± 2.1
Bioavailability	%	-	-	-	-	89.0 ± 6.1^ g^	79.0 ± 15.3	53.0 ± 22.8^ g^	57.0 ± 21.6

Data are shown as mean ± SD (*n* = 4–5). C_max_, the maximum serum concentration; T_max_, the time of C_max_; AUC_inf_, area under the serum concentration-time curve; CL, clearance for IV groups; CL/F, clearance for SC groups; V_ss_, volume of distribution at steady state for IV groups; V_ss_/F, volume of distribution at steady state for SC groups; T_1/2_, half-life. No statistical difference between lean and obese groups at corresponding dose levels and routes of administration was observed by t-tests. One-way ANOVA and post-hoc analysis was used to separately compare the parameters within the IV and SC groups. AUCinf and Cmax were normalized by dose. ^a^*p*-value: 0.0484. ^b^*p*-value: 0.0115. ^c^*p*-value: 0.0032. ^d^*p*-value: 0.0348. ^e^*p*-value: 0.0094. ^f^*p*-value: 0.0214. ^g^*p*-value: 0.0283

**Fig. 4:**
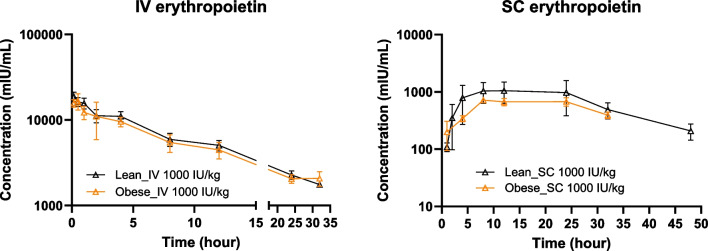
Observed pharmacokinetic profiles of human erythropoietin in obese and lean rats following IV or SC administration of 1000 IU/kg recombinant human erythropoietin (rHuEPO). Data are shown as mean ± SD (*n* = 4 per group).

Observed mean (± SD) pharmacokinetic profiles of human erythropoietin in obese and lean rats following IV or SC administration of 1000 IU/kg are shown in Fig. 4. The concentration-time profiles were very similar between lean and obese groups. Noncompartmental analysis was performed to calculate pharmacokinetic parameters as shown in Table II. No significant differences were observed between lean and obese groups except for T_1/2_ after SC dosing.

**Table II:** Pharmacokinetic Parameters for Human Erythropoietin Following Intravenous or Subcutaneous Erythropoietin Administration to Lean and Obese Rats Calculated by Noncompartmental Analysis

		IV 1000 IU/kg		SC 1000 IU/kg	
Parameter	Units	Lean	Obese	Lean	Obese
C_max_	mIU/mL	-	-	1182.9 ± 424.7	695.3 ± 99.4
T_max_	h	-	-	15.3 ± 6.9	12.0 ± 0.0
AUC_inf_	h∙IU/mL	191.1 ± 25.5	180.0 ± 38.4	38.7 ± 14.3	29.4 ± 5.9
CL or CL/F	mL/h/kg	5.3 ± 0.7	5.8 ± 1.2	30.4 ± 14.9	35.1 ± 7.7
V_ss_ or V_ss_/F	mL/kg	74.5 ± 11.1	84.7 ± 18.8	680.5 ± 472.1	1195.2 ± 191.7
T_1/2_	h	9.7 ± 0.8	10.4 ± 2.6	14.7 ± 5.0	24.5 ± 7.4 *
Bioavailability	%	-	-	20.0 ± 7.5	16.0 ± 3.3

Data are shown as mean ± SD (*n* = 4). C_max_, the maximum serum concentration; T_max_, the time of C_max_; AUC_inf_, area under the serum concentration-time curve; CL, clearance for IV groups; CL/F, clearance for SC groups; V_ss_, volume of distribution at steady state for IV groups; V_ss_/F, volume of distribution at steady state for SC groups; T_1/2_, half-life. *—significant difference between lean and obese groups (t-test, *p* < 0.05)

**Fig. 5:**
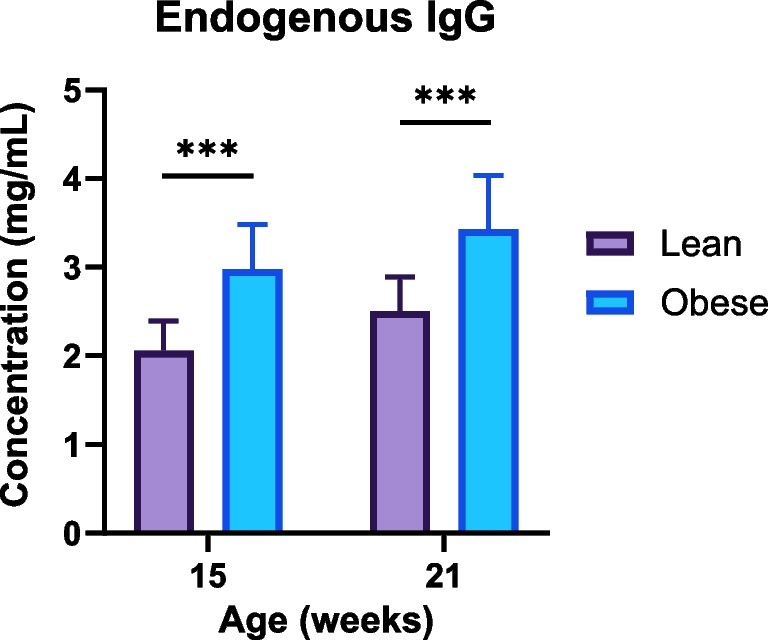
Endogenous rat IgG in obese and lean rats at the age of 15 weeks (prior to the PK study) and 21 weeks (end of the PK study). Data are shown as mean ± SD (*n* = 10 per group). ***—significant difference (t-test, *p* < 0.001).

In the nivolumab study, endogenous rat IgG in lean and obese cohorts was measured at the age of 15 weeks (before the nivolumab injection) and at 21 weeks (at the end of the study), as shown in Fig. 5. The concentration of rat IgG was statistically higher in obese rats at both times.

## Discussion

Biologics has been developed rapidly in the past decades, yet there is a lack of knowledge on biodisposition in special populations for many of them. The prevailing trend favors flat dosing, aiming to enhance medication safety and contribute to reducing of healthcare costs without compromising efficacy or safety margins [20]. Many large molecules are gaining approval as fixed-dose therapies, such as catumaxomab, obinutuzumab, ofatumumab, and pertuzumab. While initially approved by the FDA based on patient weight, pembrolizumab and nivolumab received revised approval for fixed doses [21, 22]. Flat dosing could simplify IV procedures in infusion clinics, while SC administration is increasingly explored with the potential for patients to self-administer these medications.

However, the dosing requirement in obese patients may require further investigation. A review published in 2022 summarized how obesity affected the pharmacokinetics or treatment effectiveness of biologics used to manage inflammatory bowel diseases [23]. Obesity influenced the pharmacokinetics of biologic therapies, resulting in increased clearance and reduced drug concentrations, and factors such as body weight and inflammatory markers affected drug absorption and efficacy. The review showed that the effect of obesity was more significant for adalimumab and infliximab, while the impact was less pronounced for vedolizumab and ustekinumab [23]. For example, one-third of the patients treated with adalimumab for Crohn's disease required an increase in their dosage from Q2W to QW within the first 5 months [24], and the only independent factor associated with this need for dose escalation was a higher BMI [24]. In a study of 89 infliximab-treated rheumatoid arthritis patients, obese individuals showed lower clinical response rates than non-obese patients, with 50% of those with a BMI over 30 kg/m^2^ achieving a response compared to 75% for those with a BMI between 20 and 30 kg/m^2^ and 84% for those with a BMI under 20 kg/m^2^, even after accounting for baseline disease activity and other factors [25]. In contrast, a post-hoc analysis of 254 patients treated with ustekinumab revealed no significant differences in clinical remission rates at week 44 across BMI categories, with rates of 67.9% for underweight, 51.3% for normal weight, 45.1% for overweight, and 55.3% for obese patients [26]. This suggests that the effect of obesity on treatment response may vary depending on the biologic drug.

In this study, obesity was successfully induced in animals using a high-fat diet approach, as demonstrated by significant differences in TBW, fat mass, abdominal circumference, and body length between the lean and obese groups. At the time of nivolumab dosing, the fat percentage was 17 ± 6% in obese rats compared to 7 ± 2% in control rats (Fig. 1). Previously we showed that at the age of 23 weeks (at the time of human IgG dosing) the fat percentage was 41 ± 4% in male Zucker obese rats and in comparison to 7 ± 2% in Zucker lean control rats [10]. Although the fat percentage in this diet-induced model was not as high as that observed in the genetic model of obesity, the diet-induced model offers several advantages, including the integration of genetic and dietary factors, a significant resemblance to human obesity, and cost-effectiveness [27]. Notably, a different effect of obesity on rat endogenous IgG was observed in the current study and our previous work with two genetic obesity models. In the current study, obese animals had higher endogenous IgG concentrations than lean animals, as shown in Fig. 5. In our previous work, a similar trend in rat IgG was observed in male Obese Prone (Crl:OP(CD)) and Obese Resistant (Crl:OR(CD)) rat strains [11]. Conversely, lean Zucker rats consistently had higher endogenous IgG levels than obese Zucker rats throughout the study [10]. Despite these differences, an increase in endogenous IgG concentration with age was observed in all three studies. These results indicate the importance of the selection of the animal model (strain and age) for pharmacokinetics studies of IgG-based biologics, as well as the importance of reporting endogenous rat IgG, as it might compete with exogenous antibodies (monoclonal antibodies or antibody-drug conjugates) for FcRn binding. The lack of an apparent effect of the difference in endogenous IgG between obese and lean rats on nivolumab kinetics in this study can be potentially explained by two factors. First, concentration of endogenous IgG in this study differed only by approximately 50% (while a more significant difference was observed in our other studies [10, 11]). Second, systemic concentrations of nivolumab (even after the highest dose of 8 mg/kg) were significantly lower than endogenous IgG and did not significantly alter the balance of endosomal recycling compared to much higher doses of human IgG (0.5 and 1 g/kg) in previous studies.

Our previous study found significant differences in the pharmacokinetics of human IgG after IV and SC dosing (at 1 g/kg) between Zucker obese and Zucker lean rats [10]. The AUC of human IgG in obese animals was 58% of controls after IV and 48% after SC dosing, and IgG clearance was 1.75-fold higher in obese rats [10]. In contrast with the previous study, no significant differences were identified in serum pharmacokinetic profiles of nivolumab between lean and obese groups by either intravenous or subcutaneous injection in this study. This indicates that mg/kg dosing, rather than flat dosing, is necessary to achieve equivalent exposure in obese animals. For example, assuming linear IV pharmacokinetics (as shown in this work for the studied range of doses), if obese animals had received the same flat dose of nivolumab as lean animals, the AUC in obese animals would have been 30% lower. Additionally, while no significant differences in SC pharmacokinetic profiles were observed between lean and obese rats at the corresponding dose levels, the bioavailability was inversely dependent on the dose level for both obese and lean animals. A similar dose-dependent bioavailability phenomenon was observed in our previous works for rituximab [28–30].

For most indications, a flat dosing regimen is used for nivolumab, in which it is administered as an intravenous infusion over 30 min at a dose of 240 mg every two weeks or 480 mg every four weeks. However, when combined with ipilimumab, a body weight-adjusted dosing approach is applied. In this combination therapy, nivolumab can be administered at 1 mg/kg, followed by 3 mg/kg ipilimumab on the same day every three weeks for four doses, after which nivolumab is given at 240 mg every two weeks or 480 mg every four weeks. A population pharmacokinetic model that analyzed data from 1,895 patients across 11 clinical trials found that nivolumab clearance is linear within the dose range of 0.3 to 10 mg/kg and exhibits time dependence, decreasing by up to 24.5% over time [31]. Both nivolumab clearance and the volume of distribution in the central compartment increased with body weight. However, body weight-normalized dosing provided consistent drug exposure across patients weighing between 34 and 168 kg for both Q2W and Q3W dosing regimens [31]. Current labeling for nivolumab states that body weight (ranging from 35 to 160 kg) does not significantly impact its clearance, and no dose adjustments are recommended for obese patients. Our preclinical results indicate that mg/kg dosing is essential for achieving comparable nivolumab exposure between lean and obese animals and that using flat dosing would decrease exposure in obese subjects. Further studies are needed to determine whether mg/kg dosing or flat dosing also influences the pharmacodynamic responses of nivolumab.

Currently, there are only a limited number of studies examining the pharmacokinetics of rHuEPO in the obese population. One study investigated the prevalence and predictors of epoetin hyperresponsiveness in chronic kidney disease patients to identify factors influencing variability in epoetin dosing for anemia treatment. It found a positive correlation between BMI and higher dosage requirements for epoetin alfa, suggesting that patients with a higher BMI require more epoetin to achieve the same therapeutic effect as those with a lower BMI [32]. On the other hand, a more robust response to erythropoietin was reported in obese hemodialysis patients, with lower weekly doses required to achieve the same target hemoglobin level in obese patients compared to lean individuals [33]. In a preclinical population pharmacokinetic analysis of pegylated recombinant human epoetin alfa (PEG-rHuEPO) in rats, investigators predicted that a two-fold increase in body weight would result in a 170% increase in clearance and a 238% increase in the central volume of distribution [34]. Furthermore, the analysis indicated that doubling the weight from 0.16 to 0.32 kg would decrease the estimated maximum serum concentration (C_max_) from 187 ng/mL to 104 ng/mL after subcutaneous dosing at 50 mg/kg in male rats [34], highlighting the importance of weight-based dosing to ensure adequate drug exposure. Our experimental data also showed that the serum pharmacokinetic profiles were comparable between lean and obese groups when the animals were dosed with rHuEPO according to total body weight by intravenous or subcutaneous injection.

In conclusion, the current study and existing literature suggest that obesity-induced changes in pharmacokinetics (and potentially pharmacodynamics) can vary significantly for different protein therapeutics. Furthermore, the selection of a preclinical model of obesity might affect the reported results, at least for IgG-based drugs. This emphasizes the critical need for comprehensive investigations into the pharmacokinetics of various biologics in preclinical and clinical settings. Such research is essential for optimizing dosing strategies for currently marketed and newly developed biologics, enabling effective and safe treatments tailored to special patient populations’ needs.
